# Research Progress on the Therapeutic Mechanisms of Stigmasterol for Multiple Diseases

**DOI:** 10.3390/molecules30091874

**Published:** 2025-04-23

**Authors:** Juan Li, Xinhua Zheng, Jinxu Qi

**Affiliations:** Department of Pharmacy, School of Medicine, Chongwen Campus, Pingdingshan University, Pingdingshan 467000, China; 0816@pdsu.edu.cn

**Keywords:** stigmasterol, biological activities, therapeutic potential, diseases, mechanisms

## Abstract

Stigmasterol is a plant-derived phytosterol that has attracted considerable attention because of its diverse biological activities and potential therapeutic applications. In this review, the chemical properties, biosynthesis, and biological effects of stigmasterol are exhaustively summarized. Furthermore, the anti-inflammatory, antioxidant, anticancer, neuroprotective, and hypolipidemic activities of stigmasterol have been discussed. Findings from various in vitro and in vivo studies have revealed its potential in treating various diseases, including cancer, diabetes, neurological disorders, and inflammatory conditions. The mechanisms underlying these effects are also discussed, particularly emphasizing the regulation of key signaling pathways and molecular targets, to further clarify the therapeutic role of stigmasterol. This review would provide a reference for further exploring the utility of stigmasterol as a therapeutic agent, thereby contributing to the improvement of human health.

## 1. Introduction

The search for natural compounds with unique physiological activities and pharmacological significance remains a pivotal focal point amidst the ongoing exploration in the fields of life sciences and medicine [1,2,3]. Stigmasterol is a naturally occurring steroid within the plant kingdom that transcends the boundaries of plant chemistry and is a hot research spot in the pharmaceutical field [4,5,6]. It has a distinct chemical architecture, characterized by the presence of multiple unsaturated double bonds and specific functional moieties, which confers it a wide array of biological activities [7,8]. Since its discovery as a cholesterol-lowering agent and the recognition of its growing potential in treating various maladies, its research value has become increasingly obvious [9].

In the demanding yet promising expansion of cancer research, many meticulously designed and rigorously replicated experiments have shown that stigmasterol exerts a pronounced inhibitory effect on cancer cell growth and induces apoptosis [10,11,12]. Ranging from breast to gastric cancer cells and from endometrial to ovarian cancer cells, stigmasterol manifests its singular efficacy [10,13,14]. In-depth investigations show that it can exquisitely modulate intricate signaling pathways within cancer cells, considerably influencing their proliferation, differentiation, and apoptosis [15,16,17]. For example, stigmasterol can downregulate the anti-apoptotic genes *Bcl-2* and *Bcl-xl* in breast cancer cells, thereby disrupting the anti-apoptotic equilibrium within these cells, which facilitates apoptosis [15,17]. In gastric cancer cells, it impedes the *Akt/mTOR* signaling pathway; blocking this pathway curtails cancer cell proliferation while inducing apoptosis and cytoprotective autophagy, thus impeding cancer cell growth through multiple mechanistic routes [15]. These findings highlight novel pathways for developing anticancer pharmaceuticals, enabling researchers to focus on developing more targeted, efficacious, and less toxic anticancer drugs based on the mechanisms of action of stigmasterol, thus providing new possibilities for the treatment of patients with cancer. The regulatory mechanisms of stigmasterol in glucose and lipid metabolism are gradually being elucidated in the context of the prevention and treatment of metabolic disorders. Regarding diabetes research, stigmasterol can specifically bind to the glucose transporter 4 (*GLUT4*), functioning as a conduit to facilitate glucose entry into cells, thereby substantially augmenting glucose uptake and use [18,19]. This mechanism effectively mitigates insulin resistance and enhances blood glucose regulation. It also plays a crucial role in diseases that are strongly associated with lipid metabolism, such as obesity and non-alcoholic fatty liver disease (*NAFLD*). It regulates the cardinal steps in lipid metabolism, suppressing fatty acid synthesis, and concurrently promotes oxidation and catabolism, thus reducing fat accumulation in the body, particularly in the liver [20]. These attributes make stigmasterol a potential novel therapeutic agent for diabetes and obesity, providing new perspectives and prospective solutions for the growing problem of metabolic diseases.

In-depth scrutiny of stigmasterol has significant theoretical and practical implications within the medical domain. Hence, in this review, we comprehensively summarized the biological activities, mechanisms of action, and applications of stigmasterol in the pharmaceutical industry. The aim was to provide a reference to further explore and develop this compound, thereby propelling the advancement of medical research and contributing to the improvement of human health.

## 2. Structural Characteristics, Biosynthesis, and Potential Applications of Stigmasterol

**Figure 1:**
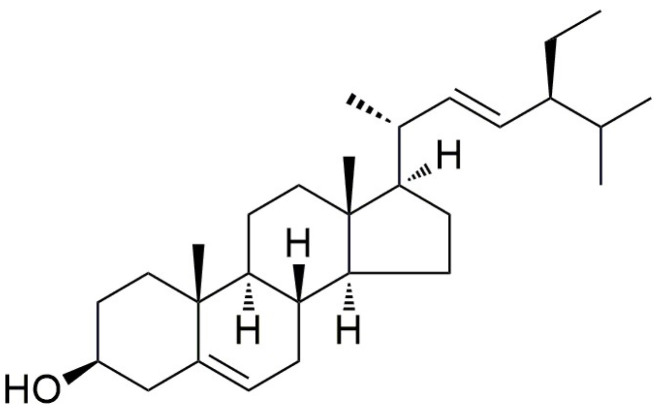
Structure of stigmasterol.

The structural feature of stigmasterol is that it is a tetracyclic triterpenoid compound, with the molecular formula of C_29_H_48_O and a molecular weight of 412.69 g/mol (Figure 1) [4,21]. Stigmasterol has a typical steroidal skeleton, which consists of three six-membered rings and one five-membered ring, and there is an ethyl side chain at the C24 position. Its biosynthetic pathway commences with the mevalonate pathway [22]. Squalene is generated through the catalysis of squalene synthase. Subsequently, with the action of oxidases and cyclases, it is transformed into cycloartenol [15,23]. Finally, stigmasterol is produced after a series of enzymatic reactions. In terms of chemical properties, stigmasterol presents as a white crystalline powder, with a melting point ranging from 161 to 170 °C, and its density is slightly higher than that of water [23,24]. It has excellent solubility in organic solvents, being readily soluble in ethyl acetate, benzene, chloroform, and pyridine, sparingly soluble in acetone and ethanol, and insoluble in water. Stigmasterol is chemically stable under normal temperature and pressure, yet it may undergo saponification or complexation reactions under acidic or alkaline conditions, which provides a chemical foundation for its application as a food emulsifier and drug carrier. In the medical field, stigmasterol exhibits anti-inflammatory, antioxidant, and anticancer activities by activating the *PPARγ* receptor and inhibiting the *NF-κB* signaling pathway [4,25,26,27]. Especially in the treatment of liver cancer, gastric cancer, and skin cancer, it demonstrates a dual effect of inhibiting tumor angiogenesis and inducing the apoptosis of cancer cells.

## 3. Anticancer Mechanisms of Stigmasterol

The high incidence and mortality rates of cancer pose a significant global health threat [28]. Dysregulated signaling pathways drive malignant proliferation, metastasis, and therapy resistance in tumor cells [29,30,31]. At the molecular level, the anti-apoptotic proteins *Bcl-2*/*Bcl-xl* enable tumor cells to evade programmed cell death by suppressing mitochondrial apoptosis, while the *Akt/mTOR* signaling pathway promotes tumor growth, metabolic reprogramming, and survival signals [32,33,34]. Akt phosphorylates pro-apoptotic proteins to enhance the anti-apoptotic function of *Bcl-2/Bcl-xl,* while mTOR activation accelerates protein synthesis to fuel tumor expansion [35,36,37]. Together, they form a core network underlying malignant progression, making their targeting a critical strategy to overcome treatment resistance.

**Figure 2:**
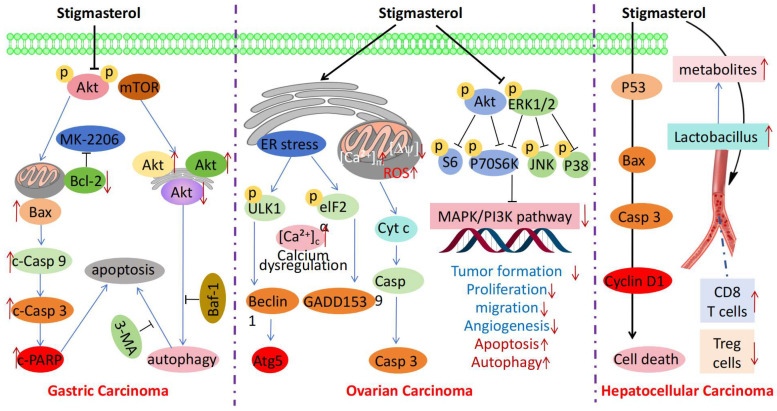
Antitumor mechanisms of stigmasterol.

Recently, stigmasterol has attracted considerable attention owing to its substantial anticancer activity (Table 1). Mojarad et al. demonstrated that stigmasterol can precisely target *Bcl-2* and *Bcl-xl* by downregulating these genes (Figure 2) [15]. This regulatory effect disrupts the antiapoptotic balance within breast cancer cells, which effectively inhibits their growth and provides a potential therapeutic approach for managing breast cancer. Zhao et al. revealed that stigmasterol effectively inhibits the *Akt/mTOR* signaling pathway, cutting off key signal transmission required for the growth and proliferation of cancer cells [16]. Additionally, it induces apoptosis and autophagy, synergistically promoting cancer cell death and inhibiting gastric cancer cell proliferation, thus offering a new direction for managing gastric cancer. Liao et al. showed that stigmasterol reduces the tolerance of cancer cells to chemotherapeutic drugs and inhibits *Nrf2* activity in patients with endometrial cancer, thereby enhancing sensitivity to chemotherapy [13]. Furthermore, Bae et al. showed that stigmasterol disrupts the normal function of the endoplasmic reticulum and mitochondria in ovarian cells, triggering a series of stress responses within the cells, which ultimately leads to cancer cell apoptosis [17]. Dong et al. found that stigmasterol regulates retinoic acid-related orphan receptor C (*RORC*) and effectively inhibits the proliferation of lung cancer cells by inhibiting *RORC* activity [10]. Additionally, it inhibits the growth and spread of lung cancer cells, which promotes apoptosis. Huo et al. pointed out that this compound reshapes the gut microbiota, regulates the balance of beneficial and harmful bacteria in the gut, and improves the gut microecological environment [38]. It can also regulate the distribution of Tregs and *CD8^+^* T cells, thereby enhancing the antitumor immune response. By modulating the function of these cells, stigmasterol disrupts the immunosuppressive state in the tumor microenvironment, thereby effectively inhibiting the growth of liver cancer cells.

## 4. Mechanisms Through Which Stigmasterol Alleviates Metabolic Diseases

The incidence of metabolic diseases such as diabetes, *NAFLD*, and obesity has rapidly increased owing to the changes in lifestyle and the accelerating aging process and has become a major public health issue [39,40,41]. Stigmasterol has gradually shown considerable therapeutic potential in this field. When insulin resistance occurs, the insulin receptor substrate (*IRS*) proteins may undergo abnormal phosphorylation, which in turn impairs the effective activation of the downstream phosphoinositide 3-kinase (*PI3K*)-protein kinase B (*Akt*) signaling pathway [42,43,44]. Consequently, the translocation of *GLUT4* to the cell membrane is affected, leading to a reduction in the cell’s uptake and utilization of glucose [42,45].

**Figure 3:**
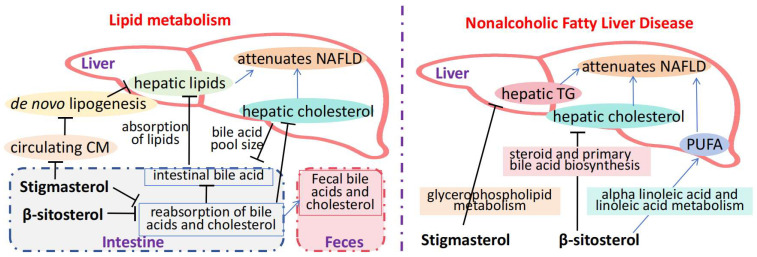
Mechanisms through which stigmasterol alleviates metabolic diseases.

In the study by Wang et al. [19], stigmasterol extracted from soybean oil exhibited antidiabetic activity in L6 cells and KK-Ay mice. It enhances GLUT4 translocation/expression, alleviating insulin resistance and glucose intolerance (Table 2). Stigmasterol was administered orally at doses of 10, 20, and 40 mg/kg for 8 weeks. The control group received an equivalent volume of vehicle solution. Glucose uptake was measured using a fluorescence-based assay, and insulin resistance was assessed via the homeostatic model assessment of insulin resistance (HOMA-IR). The results demonstrated a dose-dependent improvement in glucose uptake and insulin sensitivity, with the highest dose showing a 35% reduction in fasting blood glucose levels compared to the control group. The lipid metabolism pathway refers to a series of chemical reactions and enzyme-catalyzed processes involved in lipid synthesis, decomposition, transportation, and regulation in the human body. It includes processes such as triglyceride synthesis, cholesterol synthesis, fatty acid synthesis, β-oxidation, lipoprotein metabolism, and lipid signal transduction (Figure 3) [45,46,47,48]. Feng et al. showed that stigmasterol can regulate lipid metabolism through multiple pathways, inhibit the activities of fatty acid synthase, acetyl-CoA carboxylase, and other enzymes, reduce fatty acid synthesis, and promote the expression of related enzymes such as carnitine/organic cation transporter 2 and carnitine palmitoyltransferase 1, all of which accelerate the oxidation and breakdown of fatty acids and reduce liver triglyceride content [20,49]. Simultaneously, stigmasterol can also inhibit the activation of the nuclear factor-κB (*NF-κB*) pathway, reduce the secretion of inflammatory factors such as tumor necrosis factor-α (*TNF-α*), interleukin-6 (*IL-6*), and interleukin-1β (*IL-1β*), and alleviate liver inflammation. In animal experiments, stigmasterol reduced liver steatosis and inflammation and improved liver function indicators. Furthermore, Zhang et al. showed that stigmasterol improves lipid and bile acid metabolism, and alleviate lipid metabolism disorders, fat deposition and metabolic abnormalities induced by *HFD*. It reverses flora imbalance by reducing specific bacteria in the gut and changes serum and fecal bile acid metabolites, and the combined administration has a better effect. The mechanism is related to intestinal flora reprogramming and bile acid metabolism in enterohepatic circulation [50].

## 5. Cardiovascular Protective Mechanisms of Stigmasterol

Cardiovascular diseases are one of the leading causes of death and disability in humans globally [51,52,53]. The molecular mechanisms of cardiovascular diseases involve multiple aspects, including cellular dysfunction caused by gene mutations, disrupted signaling pathways leading to abnormal cell proliferation and differentiation, vascular endothelial damage triggered by inflammatory factors and oxidative stress, atherosclerosis caused by abnormalities in lipid metabolism-related molecules, and thrombosis resulting from an imbalance in the molecules of the coagulation and fibrinolytic systems [54,55,56]. Recently, studies have shown that stigmasterol exerts significant protective effects on the cardiovascular system. These findings provide new directions and potential strategies for preventing and treating cardiovascular diseases. Through animal experiments, Lifsey et al. demonstrated that stigmasterol promotes intestinal cholesterol excretion and reduces plasma cholesterol levels [57]. After feeding mice with stigmasterol, cholesterol levels in the bile, intestinal perfusate, and feces increased; however, plasma cholesterol levels decreased. This effect was independent of liver X receptor (*LXR*) activation. Furthermore, stigmasterol inhibits the proliferation of vascular smooth muscle cells. Li et al. revealed that this compound inhibits the proliferation of A7r5 aortic smooth muscle cells induced by angiotensin II (Ang II), thus exerting cardiovascular protective effects [58]. In vitro experiments showed that stigmasterol treatment inhibited Ang II-induced cell proliferation. Notably, stigmasterol blocks the cell cycle, promotes apoptosis, reduces reactive oxygen species (ROS) production, enhances antioxidant enzyme activity, and regulates the expression of related proteins to inhibit cell proliferation and promote apoptosis. It exerts protective effects on the cardiovascular system through these two important mechanisms and effectively prevents the occurrence and development of atherosclerosis.

## 6. Anti-Inflammatory and Immune Regulatory Mechanisms of Stigmasterol

Inflammatory responses and immune regulation play crucial roles in the complex physiological systems of the human body [59,60,61,62]. An imbalance in these two processes leads to various diseases, posing a serious threat to health [63,64,65]. Recently, studies have revealed that stigmasterol exhibits substantial anti-inflammatory and immune regulatory effects and has potential for the prevention and treatment of various inflammation-related diseases. Reportedly, stigmasterol plays a positive role in the treatment of arthritis. Inflammatory cytokines such as *TNF-α*, *IL-6*, and *IL-1β* are highly expressed during the pathogenesis of arthritis [66,67,68]. They interact to form a complex inflammatory network, which triggers inflammatory responses in the joint synovium, causing damage to joint chondrocytes, degrading the matrix, and subsequently destroying the joint structure and affecting joint function.

**Figure 4:**
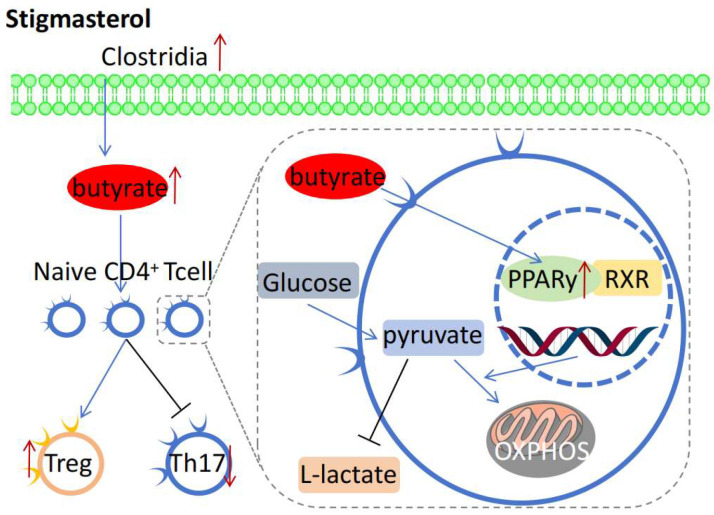
Anti-inflammatory and immune regulatory mechanisms of stigmasterol.

Zhang et al. found that stigmasterol inhibits the substance P receptor, thereby reducing airway inflammation and hyperresponsiveness in asthmatic mice [69]. In their experiment, they used ovalbumin to establish an asthmatic mouse model; after stigmasterol treatment, the expression of substance-P receptors, along with the inflammatory cell infiltration and related inflammatory factor expression, in mouse lung tissue decreased. However, lung function improved, showing that stigmasterol can potentially treat asthma. Wen et al. demonstrated that this compound alleviates intestinal inflammation by activating the butyrate-peroxisome proliferator-activated receptor γ (PPARγ) axis and restoring the balance of Treg/Th17 cells (Figure 4) [26]. They used a dextran sulfate sodium-induced mouse colitis model and found that treatment with stigmasterol increased butyrate production in the mouse gut. Furthermore, butyrate activated the *PPARγ* signaling pathway, promoting the differentiation of Treg cells and inhibiting that of Th17 cells, thereby restoring the balance of Treg/Th17 cells.

## 7. Neuroprotective Mechanisms of Stigmasterol

Neurological diseases, encompassing various conditions, severely affect the quality of life and physical health of patients [70,71,72]. The molecular mechanisms of nervous system diseases involve multiple aspects, including abnormal neuronal signaling, protein aggregation and degradation, mitochondrial dysfunction, and miRNA regulation abnormalities [73,74,75]. Recently, stigmasterol has attracted considerable attention in the field of neurological disease research. Owing to its multifaceted neuroprotective effects, this compound presents new possibilities and avenues for treating related diseases.

**Figure 5:**
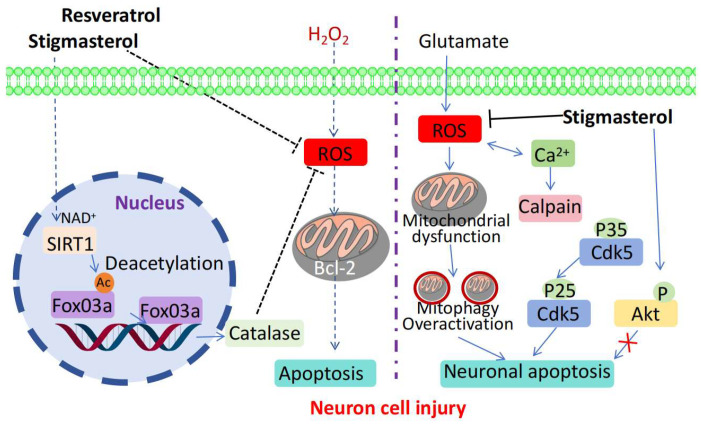
Neuroprotective mechanisms of stigmasterol.

Pratiwi R et al. found that in a model of hydrogen peroxide-induced damage in human neuronal cells, pretreatment with stigmasterol significantly inhibited the production of intracellular ROS, maintained mitochondrial membrane potential, and reduced apoptosis (Table 3) [76]. Further studies revealed that the treatment with this compound upregulated antioxidant proteins, such as forkhead box O (FoxO) 3a and catalase, as well as anti-apoptotic protein B-cell lymphoma 2 (*Bcl-2*) (Figure 5). Additionally, it activated sirtuin 1 (SIRT1), reduced acetylated lysine levels, and enhanced the antioxidant and anti-apoptotic abilities of cells. These results demonstrate that stigmasterol inhibits oxidative stress-induced neuronal cell death by modulating the expression of sirtuin family members, has a preventive and therapeutic effect against Alzheimer’s disease (AD), and represents a novel therapeutic strategy for AD. Haque et al. studied the effects of stigmasterol on neuronal migration and synapse formation [77,78,79]. In vitro neurosphere migration experiments showed that the number and distance of migrating neurones increased after stigmasterol treatment. Additionally, the expression of signaling molecules such as reelin (Reln) was increased. Molecular docking revealed that stigmasterol activates the Reln signaling pathway. Studies on hippocampal neurones have also reported that stigmasterol activates extracellular signal-regulated kinase 1/2 (*Erk1/2*) and *cAMP* response element-binding protein (*Creb*). Additionally, it upregulates actin-related protein 2 (*Arp2*) and cell division cycle 42 (*Cdc42*) and promotes synapse formation. These results demonstrate that stigmasterol promotes neuronal migration and synapse formation during neural development, which is crucial in improving cognitive function and developing new strategies for the treatment of related diseases. Liang et al. and Sun et al. studied the effects of stigmasterol on rat models of cerebral ischemia/reperfusion injury [80,81]. After stigmasterol administration, the neurological deficit scores and the cerebral infarct area decreased, and the pathological condition of the brain tissue improved. They found that stigmasterol reduced the expression of inflammatory factors such as *TNF-α*, *IL-1β*, and *IL-6*, inhibited inflammation, increased the activities of antioxidant enzymes such as superoxide dismutase (*SOD*) and glutathione peroxidase (*GSH-Px*), alleviated oxidative stress, inhibited the expression of Bax and cleaved caspase-3, upregulated *Bcl-xl*, inhibited apoptosis, modulated the *AMPK/mTOR* and c-Jun N-terminal kinase (*JNK*) signaling pathways, inhibited excessive autophagy activation, and exerted considerable neuroprotective effects. These observations highlight the potential of this compound as a therapeutic agent for cerebral ischemia/reperfusion injury. Moreover, Si et al. studied the effects of stigmasterol on a rat model of chronic constriction injury and lipopolysaccharide [27,82]. After stigmasterol administration, the symptoms of thermal and mechanical allodynia in rats were alleviated. At the molecular level, treatment with this compound inhibited the polarization of microglia to the M1 type and promoted their polarization to the M2 type. It also inhibited the expression of the components of the Toll-like receptor 4/nuclear factor-κB (*TLR4/NF-κB*) pathway, reduced the release of pro-inflammatory factors, decreased the secretion of *IL-34* by Schwann cells, inhibited the Schwann cell–macrophage cascade reaction, modulated the *IL-34/CSF1R* axis, and exerted analgesic effects. Mongkolpobsin K et al. conducted a study on the protective effects of stigmasterol against glutamate-induced neurotoxicity. In an HT-22 cell model, glutamate treatment increased intracellular ROS levels, decreased the mitochondrial membrane potential, increased apoptosis, and upregulated proteins such as *Cdk5* [83]. However, pretreatment with stigmasterol inhibited cell death, reduced ROS levels, restored the mitochondrial membrane potential, and improved autophagy. Notably, the authors found that stigmasterol alleviated neurotoxicity by enhancing *Cdk5* degradation and promoting *Akt* phosphorylation. Stigmasterol has also been combined with nanoparticles to improve its water solubility and enhance its inhibitory effect on signaling pathways, which highlights its potential in the treatment of related diseases.

## 8. Limitations, Challenges, and Future Prospects of Stigmasterol

Stigmasterol has demonstrated remarkable potential in treating diverse diseases. The therapeutic potential of stigmasterol in various diseases, as highlighted in this review, offers a promising alternative or complement to current treatment strategies. For instance, in cancer therapy, stigmasterol’s ability to downregulate *Bcl-2* and *Bcl-xl* genes and inhibit the *Akt/mTOR* pathway provides a novel mechanism for inducing apoptosis and suppressing tumor growth This contrasts with conventional chemotherapies, which often rely on cytotoxic agents that may cause significant side effects. Similarly, in metabolic disorders such as diabetes and *NAFLD*, stigmasterol’s regulation of *GLUT4* and lipid metabolism pathways offers a more holistic approach compared to existing treatments that primarily target single pathways. These findings suggest that stigmasterol could be integrated into existing treatment regimens or potentially replace certain therapies, particularly in cases where patients exhibit resistance or intolerance to conventional drugs.

One of the major obstacles is stigmasterol’s hydrophobic nature, which results in poor solubility in the body, thereby reducing its bioavailability. As a consequence, in practical applications, a higher dosage might be required to achieve the desired therapeutic effect, or specialized formulations need to be developed to enhance absorption efficiency. This not only escalates research and development costs but also limits the compound’s widespread use. Most of the existing studies have been confined to in vitro and animal experiments, with a paucity of clinical research. The absence of large-scale, multicenter clinical trial data means that its safety and efficacy in humans cannot be comprehensively verified. This lack of evidence significantly impedes the translation of research findings into clinical practice. In the context of drug development, stigmasterol’s low water solubility not only reduces bioavailability but also complicates the understanding of its metabolism and excretion processes, which in turn slows down the drug development process. Its mechanism of action is intricate, involving multiple signaling pathways such as *PPARγ* and *NF-κB*, yet the specific details within these pathways remain elusive. Furthermore, there is a dearth of adequate toxicological research. Without systematic human data, the safety of long-term use remains uncertain. The scarcity of clinical trial data also makes it difficult to gain widespread acceptance of its effectiveness in treating human diseases. Additionally, drug development must address issues such as structural optimization, the development of efficient delivery systems, and patent protection. These challenges need to be overcome to fully realize the potential of stigmasterol as a therapeutic agent.

However, the future prospects of stigmasterol are still broad. It has demonstrated significant biological activities in aspects such as anti-inflammation, anti-oxidation, anticancer, and cholesterol lowering, providing an important foundation for the development of new functional foods, drugs, and health products. With the continuous progress of nanotechnology, delivery systems, and green extraction processes, the problems of bioavailability and stability of stigmasterol are expected to be solved. At the same time, in-depth mechanism research and large-scale clinical trials will further verify its efficacy and safety, providing a scientific basis for its clinical application. In the future, stigmasterol is expected to become a new type of therapeutic drug with both low toxicity and the ability to regulate multiple targets, playing an important role in the fields of precision medicine and personalized treatment.

## 9. Conclusions

In summary, stigmasterol has remarkable therapeutic potential for various life-threatening diseases, including cancer, metabolic, cardiovascular, neurological, and inflammation-related diseases. This review provides a wealth of research directions for the development of new drugs, potentially filling the gap in treatment methods for certain diseases and improving existing treatment plans. This characteristic may contribute to the reduction in adverse reactions caused by drug toxicity and improvement of patient safety and compliance when developing related drugs or functional foods. Furthermore, stigmasterol is involved in regulating various physiological processes within the body, such as metabolism, immunity, and nervous system function, thereby maintaining the stability of the internal environment of the body and fundamentally improving body function to prevent and treat diseases caused by physiological dysfunction.

## Figures and Tables

**Table 1:** Anticancer mechanisms of stigmasterol.

Disease	Study Model	Observed Therapeutic Effects	Mechanism of Action	References
Breast Cancer	MCF-7 cell line, Balb/c mouse model of spontaneous breast tumor (SMMT)	Downregulates related genes, induces apoptosis, and inhibits cell proliferation and tumor growth	Downregulates the *Bcl-2* and *BCL-XL* genes, thereby inducing apoptosis	[15]
Gastric Cancer	SGC-7901 and MGC-803 cells, xenograft model of gastric cancer in nude mice	Inhibits cell proliferation, induces apoptosis and autophagy, and suppresses tumor growth	Inhibits *Akt/mTOR* signaling pathway, thereby inducing apoptosis and protective autophagy	[16]
Endometrial Cancer	Ishikawa cell line, related animal model (not specified in detail)	Enhances the sensitivity of cancer cells to chemotherapy	Inhibits the *Nrf2* signaling pathway, thereby enhancing the sensitivity of cancer cells to chemotherapy	[13]
Ovarian Cancer	ES2 and OV90 cells	Inhibits cell growth, induces apoptosis, ROS production, and calcium overload, and suppresses related genes	Induces endoplasmic reticulum and mitochondrial dysfunction, thereby promoting cancer cell apoptosis	[17]
Lung Cancer	Unspecified lung cancer cell lines	Inhibits cell proliferation and promotes apoptosis	Modulates retinoic acid-related orphan receptor C, thereby inhibiting cancer cell proliferation and promoting apoptosis	[10]
Liver Cancer	Subcutaneous tumor model of Balb/c mice inoculated with liver cancer cells	Suppresses tumor growth and regulates the gut microbiota and immune cell ratios	Modulates gut microbiota, thus inhibiting tumor growth	[38]

**Table 2:** Mechanisms through which stigmasterol alleviates metabolic diseases.

Disease	Study Model	Observed Therapeutic Effects	Mechanism of Action	References
Diabetes	In vitro experiments (L6 cells), KK-Ay mice	Promotes glucose uptake and improves insulin resistance and blood glucose indices	Targets the *GLUT4* transporter and regulates its expression and translocation	[19]
Nonalcoholic fatty liver disease (NAFLD)	Mouse model of NAFLD induced by a high-fat diet	Reduces hepatic steatosis and improves lipid metabolism indices	Inhibition of *NF-κB* pathway alleviates hepatitis and steatosis	[45,46,47,48]
HFD—induced dyslipidemia, obesity, hepatic steatosis	Rats on HFD, some given ST, fecal microbiota transplant tests, combined—treatment tests	Relieve HFD lipid disorder and improve treatment with combined medication	Reverse the imbalance of flora, change BA metabolism, associated with enterohepatic circulation	[50]

**Table 3:** Neuroprotective Mechanisms of Stigmasterol.

Disease	Study Model	Observed Therapeutic Effects	Mechanism of Action	References
Alzheimer’s disease	Human neuronal cells (SH-SY5Y cells)	Upregulated FoxO 3a, catalase, Bcl-2, increased SIRT1 expression, decreased acetylated lysine levels, and stimulated SIRT1 activity.	Controlling ROS, promoting anti-oxidative and anti-apoptotic factors, and activating SIRT1 through lysine de-acetylation.	[76,77,78,79]
Cerebral Ischemia/Reperfusion Injury	Rat model of cerebral ischemia/reperfusion injury	Reduces damage, improves pathological changes, and inhibits apoptosis	Reduces oxidative stress and inflammation and inhibits autophagy	[80,81]
Neuropathic Pain	Rats with CCI and cell cultures (microglia, Schwann cells, macrophages)	Reduced pain hypersensitivity in rats. Altered cytokine levels. Regulated M1/M2 polarization markers. Lowered IL-34, CSF1R, NLRP3 levels in relevant cells and tissues.	Regulates microglial M1/M2 polarization through the *TLR4/NF-κB* pathway. Reduces *IL-34*-CSF1R-mediated activation and NLRP3 inflammasome activation.	[82]
Glutamate-Induced Neurotoxicity	HT-22 cells	Inhibits cell death, regulates related indicators, and downregulates protein expression	Downregulates the *Cdk5/p35/p25* signaling pathway	[83]

## Data Availability

Data are contained within the article.
